# Broad HIV Epitope Specificity and Viral Inhibition Induced by Multigenic HIV-1 Adenovirus Subtype 35 Vector Vaccine in Healthy Uninfected Adults

**DOI:** 10.1371/journal.pone.0090378

**Published:** 2014-03-07

**Authors:** Jakub Kopycinski, Peter Hayes, Ambreen Ashraf, Hannah Cheeseman, Francesco Lala, Justyna Czyzewska-Khan, Aggeliki Spentzou, Dilbinder K. Gill, Michael C. Keefer, Jean-Louis Excler, Patricia Fast, Josephine Cox, Jill Gilmour

**Affiliations:** 1 International AIDS Vaccine Initiative (IAVI), Human Immunology Laboratory, Imperial College, London, United Kingdom; 2 University of Rochester School of Medicine and Dentistry, Rochester, New York, United States of America; 3 IAVI, New York, New York, United States of America; Imperial College, United Kingdom

## Abstract

A correlation between *in vivo* and *in vitro* virus control mediated by CD8+ T-cell populations has been demonstrated by CD8 T-cell-mediated inhibition of HIV-1 and SIV replication *in vitro* in peripheral blood mononuclear cells (PBMCs) from infected humans and non-human primates (NHPs), respectively. Here, the breadth and specificity of T-cell responses induced following vaccination with replication-defective adenovirus serotype 35 (Ad35) vectors containing a fusion protein of Gag, reverse transcriptase (RT), Integrase (Int) and Nef (Ad35-GRIN) and Env (Ad35-ENV), derived from HIV-1 subtype A isolates, was assessed in 25 individuals. The vaccine induced responses to a median of 4 epitopes per vaccinee. We correlated the CD8 responses to conserved vs. variable regions with the ability to inhibit a panel of 7 HIV-1 isolates representing multiple clades in a virus inhibition assay (VIA). The results indicate that targeting immunodominant responses to highly conserved regions of the HIV-1 proteome may result in an increased ability to inhibit multiple clades of HIV-1 *in vitro*. The data further validate the use of the VIA to screen and select future HIV vaccine candidates. Moreover, our data suggest that future T cell-focused vaccine design should aim to induce immunodominant responses to highly conserved regions of the virus.

## Introduction

CD8 T-cells have been shown to effectively control HIV replication *in vivo* [1] and in both SIV infection and vaccinated SIV-challenged NHP [2], [3]. The induction of broadly cross-reactive, anti-viral T cells is likely to be a crucial facet of an efficacious HIV vaccine [4], which would complement humoral responses containing the virus at the site of infection. The breadth of HIV-1 p24-specific CD8 T-cell responses has previously been associated with control of virus replication in HIV-infected individuals [1], [5], [6]. Narrowing virus-specific responses to fewer regions may increase the chance of epitope escape by limiting the selective pressure imposed upon the virus [7]. Furthermore, the focusing of responses towards more variable regions of the HIV proteome following vaccination limits the likelihood that a transmitted founder virus might be recognized and controlled, one potential explanation for the failure of the STEP Ad5 and HVTN505 trials [8]–[10]. As a consequence, it is crucial to determine the breadth and specificity of vaccine-induced T-cell responses through epitope mapping and functional viral inhibition assays (VIA) using a broad panel of viruses.

This study focuses on insert-specific cellular responses induced following vaccination, with a view to assessing the potential ability of such responses to limit virus replication, thereby lowering set-point and potentially decreasing the likelihood of virus transmission. Previous HIV vaccine trials such as the STEP Ad5 study relied primarily on validated assays, IFNγ, using pools of peptides to assess vaccine T-cell immunogenicity including rate of vaccine take, magnitude and duration of responses [11], [12]. Both assays predominantly focus on the production of IFNγ by insert-specific T-cell populations. The majority of HIV-infected individuals are capable of generating long-lasting, HIV-specific T-cell responses, however only a small proportion of these are able to limit the acute virus burst during early infection and control virus replication in the long term [1], [13], [14]. Such responses are also usually measured by the production of IFNγ and use high concentrations of exogenously added peptides. The measurement of IFNγ largely fails to correlate with protection [15] and so vaccine trials lack a way of assessing whether vaccine-specific immune responses would be potentially capable of controlling virus replication *in vivo*. We and others have shown that an *in vitro* viral inhibition assay correlates well with *in vivo* virus control and may help characterise vaccine-induced T-cell responses when coupled with epitope mapping [16]–[19].

Future vaccine candidates should induce responses capable of targeting and inhibiting multiple virus isolates across multiple clades, thereby overcoming the hurdle of virus sequence variability. It is therefore prudent to assess any potential vaccine candidate in its ability to elicit virus inhibition activity. Co-culture of HIV infected CD4 cells with autologous virus-specific CD8 populations has previously been shown to result in *in vitro* virus inhibition as measured by the reduction in HIV p24 in supernatants [20], [21]. More recently, VIA has been shown to correlate with virus control in vaccinated and challenged NHP [2]. Others have shown that VIA activity in the draining lymph nodes of NHP, following vaccination with live attenuated SIV, correlates with sterilizing protection [22], [23]. VIA data generated by Freel *et al*. [24] demonstrate that vaccine-induced responses can inhibit a multi-clade panel of viruses; in their study the production of MIP1-β production along with degranulation, as determined CD107a accumulation, by CD8+ T-cell populations was shown to be a predictor of virus inhibition. Yang *et al*. [19] demonstrated that the presence of virus-specific CD8 T-cells during acute infection capable of *in vitro* inhibition of HIV-1 was associated with a delay in the rate of CD4 decline; the induction of any such responses following vaccination would be highly desirable.

In the present study, in addition to IFNγ ELISpot and ICS assays, vaccine recipients immunized with recombinant Ad35-GRIN and Ad35-Env were assessed for their ability to inhibit a panel of HIV viruses *in vitro*, using the VIA [16]. The magnitude, breadth and specificity of insert specific T-cell responses were initially assessed using peptide pools corresponding to the insert-matched Gag, RT, Int, Nef and Env antigens, using a validated IFNγ ELISpot assay [25], [26]. Peptide matrix pools were subsequently designed and the ELISpot assay further qualified to allow the deconvolution of individual peptides within the responding antigen pools. This, in combination with information on the individual HLA phenotype of the subjects and published datasets on previously defined HIV epitopes (obtained using the LANL HIV immunology database www.hiv.lanl.gov), permitted the simultaneous resolution of whether the vaccine-induced responses were targeted against multiple regions of the insert and whether those regions were conserved because of functional importance to the virus. Furthermore, by assessing the degree to which immunodominant putative CD8 epitopes recognized following vaccination are conserved relative to the individual viruses used in the VIA panel, we attempted to discern whether responses to more conserved regions of HIV would be better able to control a range of possible virus variants and whether the targeting of certain regions was advantageous over others. These findings might have implication for rational vaccine design-to induce a broadly cross reactive and potent anti-viral T cell response.

## Methods

### Trial Participants

Healthy HIV-uninfected male and female adults aged 18–50 years were recruited at the University of Rochester, NY, USA. Volunteers reported low-risk behavior for HIV (i.e., no unprotected vaginal or anal sex with known HIV-infected person; no sex in exchange for money or drugs; no sexually transmitted infection within 6 months before enrollment) and they were willing to undergo HIV testing and receive results. Sexually active women agreed to use effective contraceptive methods at least until 4 months after the second vaccination. Only subjects without baseline serum neutralizing antibodies against Ad35 were enrolled [27].

### Ethics Statement

This study was approved by the Western Institutional Review Board (WIRB). The study was conducted in accordance with International Conference on Harmonization - Good Clinical Practice (ICH-GCP) and Good Clinical Laboratory Practice (GCLP). All participants provided written informed consent.

### Vaccine description/schedule

The phase I randomized, double-blind, dose-escalation, placebo-controlled HIV-1 vaccine trial was conducted to assess the safety and immunogenicity of escalating doses of two recombinant replication-deficient adenovirus serotype 35 (Ad35) vectors containing HIV gag, RT, int and nef (Ad35-GRIN) and env (Ad35-ENV) [27] (*Trial Registration:* ClinicalTrials.gov NCT00851383, IAVI B001). Individuals in Groups A, B and C received intramuscular vaccinations with a 1∶1 mixture of Ad35 GRIN and Ad35 ENV constructs at 0 and 6 months at either 2×10^9^, 2×10^10^ or 2×10^11^ viral particles. Individuals in Group D received vaccinations with only Ad35-GRIN at 1×10^10^ viral particles, omitting the Ad35 ENV construct. HIV-specific cellular responses were seen in the majority of vaccinated volunteers, with reactogenicity increasing with dose and upon subsequent boosting vaccination. Polyfunctional T cell responses were capable of being induced in both either CD4 and/or CD8 populations against all inserted antigens [27].

### PBMC sample preparation and HLA typing

PBMC were isolated using density gradient separation from heparinized whole blood, frozen in a mixture of fetal bovine serum (Sigma-Aldrich, St Louis, MO) and DMSO (9∶1 ratio) using a Kryo 560-16 rate controlled freezer (Planer, Sunbury-On-Thames, UK). PBMC were stored and shipped in vapor phase liquid nitrogen to the IAVI Human Immunology Laboratory (HIL), Imperial College, London [25]. HLA typing was performed to 4-digit resolution, using PCR with sequence-specific primers (IMGM Laboratories GmbH, Martinsried – Germany).

### ELISpot Assay

An ELISpot assay validated for use in assessing vaccine-induced T-cell responses was employed, described previously [25], [26]. In brief, pre-coated IFNγ ELISpot 96-well plates (Mabtech AB, Nacka Strand, Sweden) were washed 3 times with 200 µl PBS (Sigma, Dorset, UK) prior to blocking with 200 µL R10 media (RPMI 1640) supplemented with 10% (v/v) fetal bovine serum (FBS) 2 mM L-glutamine, 100 units penicillin, 0.1 mg/mL streptomycin, 10 mM HEPES buffer and 1 mM sodium pyruvate (all from Sigma) and incubated at 37°C for a minimum of 2 hours. Cryopreserved PBMC were thawed, washed and resuspended in R20 (as for R10 but supplemented with 20% v/v FBS) and incubated overnight in a humidified incubator at 37°C with 5% CO_2_ in air. On the day of the assay, cells were counted (Vi-cell counter, Beckman Coulter) and resuspended in R10 at 4×10^6^ viable cells/mL. Blocking R10 media was decanted and 100 µL of peptide (1.5 µg/mL final concentration), PHA or media control were added followed by 50 µL of cells to give a density of 200,000 cells/well. Plates were incubated as above for 16–24 hours. Six HIV-1 peptide pools one pool each representing Gag (125 peptides), Pol/Int (102 peptides), RT(111 peptides), Nef (49 peptides) and 2 pools representing the Env sequence (total 158 peptides) were used in the ELISpot assay. The pools consisted of 15 mers with 11 overlapping amino acids, the individual peptides were 15 mers synthesized by AnaSpec, Inc. (Fremont, CA, USA) to 90% purity. Spot forming cells (SFC) were counted using an automated AID ELISPOT reader (Autoimmun Diagnostika, Strassberg, Germany).

### Peptide Mapping

Individuals with background-subtracted IFNγ ELISpot responses of >100 SFU/million PBMC were selected for epitope mapping. The epitope mapping matrix pools were designed using Deconvolute This! Software v1.0 (kindly provided by M. Roederer NIH) [28]. Each matrix pool consisted of 10 insert-matched 15mer peptides, with a sequential 11 amino acid overlap, which correspond to each antigen in three different configurations. Positive responses were described as being >5x background and >50 SFU/million. Positive wells were used to deconvolute potential positive peptides through Deconvolute This! software. ELISpot responses to mapped peptides were retrospectively related to their whole antigen-specific T-cell lineage response, as determined by flow cytometry. Putative epitopes were assigned based on the HLA background of the vaccinees with previously described epitopes found in the LANL database.

### Flow Cytometry

Flow cytometry was performed as described previously [27]. Briefly, antigen-specific phenotypes and cytokine secretion profiles were assessed using a qualified polychromatic flow cytometry (PFC) panel. PBMC were co-incubated with peptide pools matched to the GRIN/ENV insert, 1 µg/ml SEB (Sigma-Aldrich, St. Louis, MO, USA) or mock stimuli, CD107a PECy5, BD Golgistop (Becton Dickinson, San Jose, CA, USA) and Brefeldin A (Sigma-Aldrich, Poole Dorset, UK) for 6 hours at 37°C. Cells were stained for viability with LIVE/DEAD® Fixable Violet Dead Cell Stain Kit (Invitrogen, Eugene, OR, USA), and then surface stained by anti-CD4 QD605, anti-CD8 pacific orange, anti-CD19 pacific blue (Invitrogen, Paisley, UK), anti-CD27 APC-H7, anti-CD14 pacific blue, anti-CD57 FITC, anti-B7 integrin PE (Becton Dickinson, San Jose, CA), and anti-CD45RO ECD (Beckman Coulter, High Wycombe, UK). Finally cells were stained intracellularly with anti-CD3 QD655 (Invitrogen, Paisley, UK), anti-IFNγ PE Cy7, anti-TNF-α A700 and anti-IL-2 APC (Becton Dickinson, San Jose, CA, USA) washed and acquired on the same day. At least 750,000 events were acquired on a custom-built BD LSR II cytometer. Data were analyzed and presented using FlowJo (version 8.8 Treestar).

### Viral inhibition assay

A VIA assay qualified for use in vaccine trials as described below was used [16].

#### Generation of CD4+ target and CD8+ effector T cells

PBMCs were resuspended at a density of 1×10^6^ cells/mL in R10 medium supplemented with 50 U of IL-2 and 0.5 µg/mL CD3/CD4 or CD3/CD8 bispecific antibodies (a generous gift from Johnson Wong, Harvard Medical School) for generation of CD8 or CD4 T cells, respectively [29]–[31]. Culture volumes were doubled at days 3 and 6 by addition of fresh medium and IL-2. CD4 T cells were infected, at a multiplicity of infection (MOI) of 0.01, for 3 h with a panel of exogenous HIV-1 isolates – IIIB (subtype B), ELI (accession number A07108, subtype B), U455 (M62320 subtype A), and 97ZA012 (AF286227, subtype C) (provided by the HIV AIDS reagent repository), CH77 (FJ496000, subtype B), CH106 (NA, subtype B), 247FV2 (FJ496200, subtype C) (generously donated by George Shaw, University of Birmingham, Alabama).

Culture conditions, as described previously [16], were standardized across all inhibition experiments; 0.5×10^6^ exogenously infected (MOI, 0.01) 7-day antibody-expanded CD4+ T cells were cultured with or without 0.5×10^6^ autologous 7-day antibody-expanded CD8+ T cells in 1 mL of medium with 50 U of IL-2 in 48-well plates. Half of the well supernatant was replaced with medium and IL-2 on days 3, 6, 8, and 10. Supernatant p24 content was measured on day 13 by enzyme-linked immunosorbent assay (ELISA) (PerkinElmer). CD8+ T-cell-mediated inhibition was expressed as the log_10_ reduction in p24 content of day 13 CD8+ and CD4+ T-cell co-cultures, compared with infected CD4+ T cells alone. For clinical trial subjects, antibody-expanded pre-vaccination CD4+ T cells were used as common targets for HIV-1 infection in co-cultures with pre- and post-vaccination CD8+ T cells. Where possible the maintenance of antigen pool-specific responses following expansion was assessed by IFNγ using both expanded T-cell subsets. The threshold used for positive inhibition was determined from previous validation studies as reduction in measurable p24 production of >1.5 logs.

### Determination of relative conservation of antigenic sequences

Following a sequential alignment between the vaccine insert and each virus used in the panel using the QuickAlign tool on the Los Alamos database (www.hiv.lanl.gov), degree of conservation towards vaccine-induced epitopes was deduced relative to analogous sequences in the panel of viruses. HeatMaps with hierarchical clustering were made using LANL software. (http://www.hiv.lanl.gov/content/sequence/HEATMAP/heatmap.html)

### Statistical Analysis

All statistical analyses were performed using Prism 5.0a (GraphPad Software, Inc.La Jolla, CA USA). To determine whether there was a relationship between the degree of virus inhibition and the relative conservation of epitopes targeted, a linear regression analysis was performed using a 95% confidence interval. To assess whether the degree of inhibition was related to the conservation of sequences targeted, a Mann Whitney t-tests for unmatched pairs were performed by assessing the inhibition of a virus where the putative epitope was conserved compared to where it was not conserved. Graphs illustrating functional phenotypes of responding cells were produced using Pestle V1.7 (donated by M.Roederer) and SPICE Version 5.3 downloaded from http://exon.niaid.nih.gov [32].

## Results

### ELISpot Responses

Cellular immune responses were assessed using cryopreserved PBMC collected at enrolment and at 2 and 4 weeks post first and booster vaccination. 86% of vaccinees had responses to at least one of the vaccine antigens at two weeks post-booster vaccination. For each antigen, individuals with pool-specific screening ELISpot responses >100 SFU/million were selected for epitope mapping using corresponding peptide matrix. Of 56 vaccinees, 25 fulfilled the threshold selection criterion and were selected for insert pool-specific mapping. A total of 54 peptide matrices were used across all volunteers and antigen pools. In all but two vaccinees, a cut-off of >50 SFU/million was applied (following background subtraction) for peptide matrices in order to deconvolute potential responses. The two exceptions had responses >40 SFU/million following mock subtraction and were the only responding pools in the matrix. Of all mapped vaccinees, 56% mounted responses to RT, 48% responded to either Gag and/or Env, 40% responded to Int and 24% responded to Nef (Table 1). Of those vaccinated with Ad35 containing the Env insert (Groups A–C), 71% mounted responses to this antigen. This suggests that Env is immunodominant when compared to the other antigens and to Group D who received only Ad35-GRIN.

**Table 1:** Breadth of peptides recognized following vaccination with Ad35-GRIN and Ad35-ENV.

Antigen	Number of peptides mapped	Minimum number of peptides mapped per vaccinee	Maximum number of peptides mapped per vaccinee	Mean	Median	Total number of epitopes mapped
GAG	12	1	3	1.5	1	18
RT	14	1	3	1.79	2	25
INT	10	1	4	1.8	1	18
NEF	6	1	2	1.17	1	7
ENV	12	1	4	2.08	2	25
TOTAL	25	1	9	3.72	4	93

**Figure 1:**
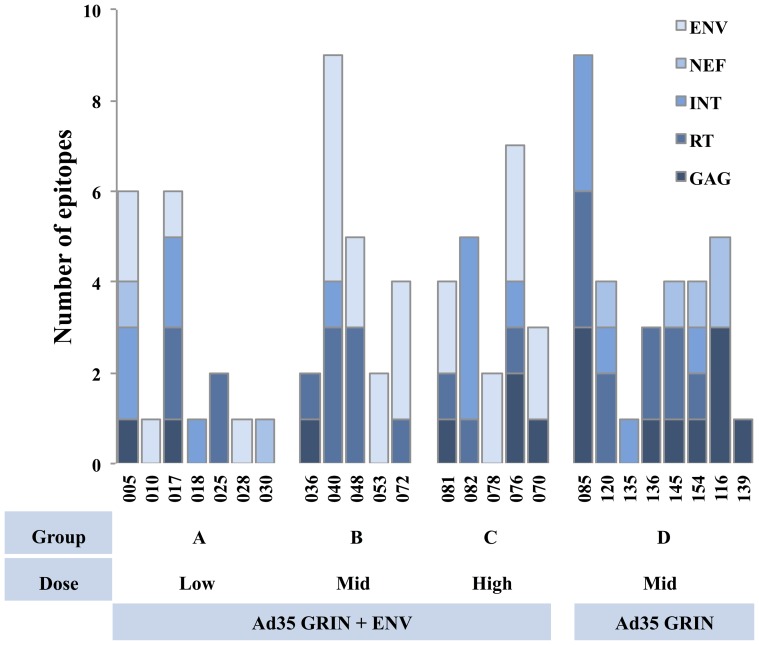
Breadth of insert-specific T-cell responses. Elispot peptide matrices were used to determine the number of possible epitopes recognized by vaccinees. Vaccinees (classified by Volunteer IDs) are stratified by dose and vaccine regimen.

Figure 1 shows the breadth of pool specific T-cell responses for each vaccinee stratified by dose and vaccine regimen. The mean number of epitopes recognised per participant was 3.72, with a median of 4 and a range of 1–9. Where possible, responding peptide sequences were subsequently related to responding T-cell lineages, as ascertained through ICS of antigen pool-specific populations [27]. 29 samples were assessed by ICS and/or ELISpot, and responses were predominantly in CD8+ populations. We then determined whether the responses were associated with previously defined optimal epitopes and the volunteer's specific HLA alleles. Table S1 (in File S1) lists all the proteins targeted and the epitopes/peptides recognized by each vaccinee, Table 2 then lists the immunodominant putative CD8 T-cell epitopes matched with the vaccinee's HLA background.

**Table 2:** Reactive peptides inducing responses in CD8 populations ascertained through ICS and ELISpot using expanded CD8 populations, where possible peptides are associated with predefined optimal epitopes using vaccinee HLA.

VID	HLA-A	HLA-A	HLA-B	HLA-B	HLA-C	HLA-C	Antigen	Peptide	Putative epitope	HLA Associations
085	A*02:01	A*02:01	B*44:02	B*44:02	C*05:01	C*05:01	INT	EDHERYHSNWR	EDHERYHSNW	B*44:03 epitope
116	A*01:01	A*02:01	B*08:01	B*35:03	C*04:01	C*07:01	NEF	EEEEVGFPVR	EEVGFPVR	A*02
120	A*24:02	A*29:02	B*45:01	B*55:01	C*03:03	C*06:02	NEF	EEEEVGFPVR	EEVGFPVR	B*45
040	A*02:01	A*02:01	B*07:02	B*58:01	C*07:02	C*07:18	INT	EFGIPYNPQSQGVVA	IPYNPQSQGV	B*07
076	A*33:03	A*66:01	B*15:10	B*78:01	C*03:04	C*16:01	RT	ETFYVDGAANR	ETFYVDGAANR	A*66
018	A*01:01	A*02:05	B*50:01	B*51:08	C*06:02	C*16:02	INT	FNLPPIVAKEI	LPPIVAKEI	B*51
082	A*02:01	A*02:01	B*39:01	B*51:01	C*02:02	C*07:02	INT	FNLPPIVAKEI	LPPIVAKEI	B*51
135	A*02:01	A*24:02	B*07:02	B*51:01	C*01:02	C*07:02	INT	FNLPPIVAKEI	LPPIVAKEI	B*51
136	A*02:01	A*68:01	B*40:01	B*44:02	C*03:04	C*05:01	RT	HRTKIEELRAHLLSW	KIEELRAHL	A*02
085	A*02:01	A*02:01	B*44:02	B*44:02	C*05:01	C*05:01	RT	IEELRAHLLSW	KIEELRAHL	A*02, B*44
116	A*01:01	A*02:01	B*08:01	B*35:03	C*04:01	C*07:01	NEF	IWKFDSRLALK	WKFDSRLALK	A*01
085	A*02:01	A*02:01	B*44:02	B*44:02	C*05:01	C*05:01	GAG	KALRAEQATQDVKGW	AEQATQDVKGW	B*44:02
040	A*02:01	A*02:01	B*07:02	B*58:01	C*07:02	C*07:18	RT	KGSPAIFQSSM	SPAIFQSSM	B*07
040	A*02:01	A*02:01	B*07:02	B*58:01	C*07:02	C*07:18	RT	KVAMESIVIWGKTPK	KVAMESIVIW	B*57/58 analogue
085	A*02:01	A*02:01	B*44:02	B*44:02	C*05:01	C*05:01	GAG	LFNTVATLYCV	LFNTVATLY	A*02
048	A*02:01	A*26:09	B*13:02	B*38:01	C*06:02	C*12:03	ENV	MHEDIISLWDQ	MHEDIISLW	A*02, B*38
053	A*02:01	A*26:01	B*07:02	B*38:01	C*07:02	C*12:03	ENV	MHEDIISLWDQSLKP	MHEDIISLW	A*02, B*38
048	A*02:01	A*26:09	B*13:02	B*38:01	C*06:02	C*12:03	RT	QGQDQWTYQIYQ	GQDQWTYQI	B*13
030	A*01:01	A*24:02	B*07:02	B*18:01	C*07:01	C*07:02	NEF	REVLIWKFDSRLALK	WKFDSRLALK	A*01
081	A*03:01	A*24:02	B*14:02	B*35:02	C*04:01	C*08:02	ENV	RYLRDQQLLGI	RYLRDQQL	A*24:02
076	A*33:03	A*66:01	B*15:10	B*78:01	C*03:04	C*16:01	ENV	SNLLRAIEAQQQLLK	RAIEAQQQLL	B*15, Cw*03:04
025	A*03:01	A*74:01	B*15:03	B*18:01	C*02:10	C*05:01	RT	STNNETPGVRY	NNETPGVRY	B*18
116	A*01:01	A*02:01	B*08:01	B*35:03	C*04:01	C*07:01	GAG	VGNIYKRWIILGLNK	NIYKRWII	A*02, B*08
040	A*02:01	A*02:01	B*07:02	B*58:01	C*07:02	C*07:18	RT	VQPIMLPDKESW	IMLPDKESW	B*58:01
025	A*03:01	A*74:01	B*15:03	B*18:01	C*02:10	C*05:01	RT	WASQIYAGIKVKQLC	QIYAGIKVK	A*03
036	A*01:01	A*03:01	B*27:05	B*57:01	C*01:02	C*06:02	RT	WASQIYAGIKVKQLC	QIYAGIKVK	A*03
072	A*03:01	A*11:01	B*08:01	B*13:02	C*06:02	C*07:01	RT	WASQIYAGIKVKQLC	QIYAGIKVK	A*03
036	A*01:01	A*03:01	B*27:05	B*57:01	C*01:02	C*06:02	GAG	YKRWIILGLNK	KRWIILGLNK	B*27:05
139	A*11:01	A*29:02	B*27:05	B*44:03	C*02:02	C*16:01	GAG	YKRWIILGLNK	KRWIILGLNK	B*27:05

Bold sequences appear more than once and indicate a preferential immune targeting.

### Immunodominance and conservation of regions targeted

Table 1 and figures S1–4 (in file S1) show the peptide sequences targeted and the number of vaccinees recognising each antigen within the vaccine insert. Gag-specific responses were found predominantly in three regions of p17, three regions of p24 and one region of p2p7p1p6. Five out of twelve vaccinees that responded to Gag recognised the peptide containing the DRFALNPSLLE epitope, while 3/12 recognised the DAWEKIRLRPG peptide sequence, both present in p17. Three individuals recognized the highly conserved peptide containing the B*2703 KRWIILGLNK epitope, found within the p24 region of Gag (Figure S1 in file S1). Two of these vaccinees expressed the B*2703 allele, the other likely recognized the upstream NIYKRWII analogue of the EIYKRWII epitope previously shown to be recognized by both B*08 and A*0201 HLA types, both present in this individual. The majority of vaccine-induced responses were directed against the p17 region, which tends to have higher variability than p24.

The mapped RT and Int specific responses were combined for analysis; with more than 20 distinct regions in Pol being recognized compared to 7 in Gag (Figure S1 and S2 in file S1). The most immunogenic region recognized was within the RT sequence WASQIYPGIKVRQLC, containing epitopes recognized by HLA A*03, A*11, A*30 and B*42 alleles. 5/12 RT responding vaccinees expressed either A*30, A*03 and/or A*11 alleles. Those individuals expressing the HLA A*0301 allele targeted their immunodominant responses towards the QIYAGIKVR epitope contained within this peptide. Furthermore, participants expressing the HLA B*51 allele all targeted immunodominant responses against the LPPIVAKEI epitope. The most conserved region targeted within Pol was YNVLPQGWKGSPAIFQSSM sequence, this contained epitopes recognised by 2/12 individuals, while 3/12 individuals reacted to the highly variable region containing the ESIVIWGKTPK sequence, which has not previously been assigned to either HLA class I or II binding alleles.

When present in the Ad35 GRIN plus Env vaccine regimen, ENV was able to induce a response above the selection threshold in the majority of selected vaccines, also inducing a greater response breadth than the other antigens. Six out of twelve (50%) vaccinees responded to PCRIKQIIRMW, although none expressed the HLA alleles normally associated with epitopes previously defined within this sequence and all of these responses were of a low magnitude (<150 SFU/million). However, 2 vaccinees mounted immunodominant responses towards the conserved MHEDIISLWDQ sequence containing known HLA A*02 and B*38 epitopes, both individuals expressed these alleles.

Nef specific responses were relatively infrequent with only 6/25 vaccinees mounting responses above the selection threshold. However, following deconvolution, 4/6 peptide-specific responses were found to be over 200 SFU/million and were focused towards one of two regions that have been previously identified as being epitope-rich. The association of the optimal epitopes with vaccinee HLA was difficult to resolve due to the innate variability of the sequences targeted.

By focusing on previously defined epitopes, which induced responses only in the CD8+ T-cell populations, and by imposing a further 150 SFC/million threshold, we were able to limit analysis to immunodominant CD8+ epitopes. By imposing these restrictions a trend towards the preferential targeting of the epitopes by individuals expressing particular HLA types such as A*02, A*03, B*27, B*38 and B*51 (see table 2) could be clearly seen, indicating vaccine responses may, in certain cases, be predicted and therefore the potential exists for immune focusing of T-cell responses in future vaccine inserts.

### Virus Inhibition Assay (VIA)

**Figure 2:**
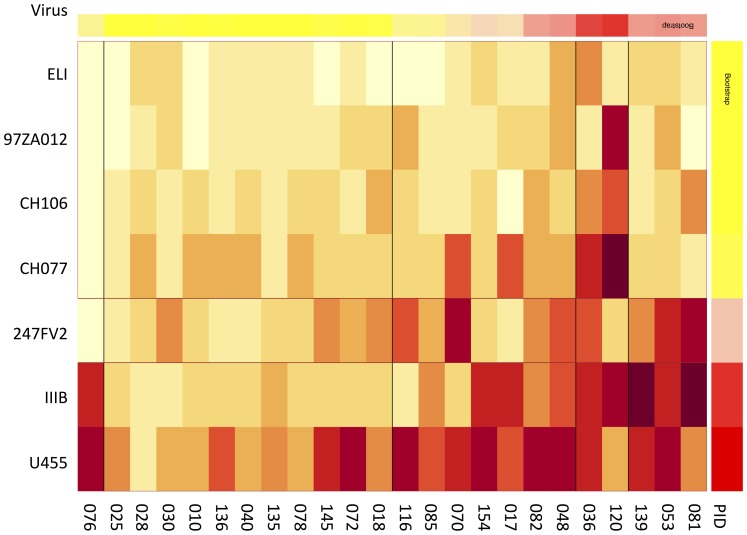
Ability of vaccine-induced CD8 responses induced to inhibit multiple virus isolates. Heatmap illustrating the degree of virus inhibition of a cross-clade panel of viruses by vaccinees. The darker the colouring, the higher the inhibition. Associated table S3 (in file S1) illustrates virus inhibition range and number of viruses inhibited.

The efficacy of vaccine-induced HIV-specific CD8+ T cells to inhibit HIV replication *in vitro* was measured against a panel of HIV-1 isolates. Of the epitope mapped individuals, most were able to inhibit U455 (subtype A) by more than 1.5 logs, this was followed by IIIB (subtype B) >247FV2 (subtype C) >CH077 (subtype B) >CH106, 20.8% >(clade B, as shown in Figure 2). When assessing the comparative sequence identity between all viruses used in the panel, there was no obvious trend associating overall amino acid sequence identity between viruses and the insert and the average capacity of vaccinees to inhibit viruses (Table 3). Although 83% of vaccinees inhibited IIIB, the shared sequence identity between IIIB and the vaccine insert was low. The frequency of inhibition was related to the average levels of inhibition.

**Table 3:** Amino acid % sequence identity of vaccine insert compared with virus inhibited in VIA.

Virus	Accession No.	Clade	GAG	POL	NEF	ENV	Total	Av. VIA
U455	M62320	A	87.3	94.2	75.9	82.4	85.0	3.10
ELI	A07108	D	83.6	90.8	77.7	75.0	81.8	0.88
IIIB	K03455	B	81.4	91.3	77.2	73.0	80.7	2.36
CH77	FJ496000	B	82.1	90.2	74.5	72.8	79.9	1.77
CH106	JN944942	B	81.8	90.4	78.0	72.5	80.7	1.37
247FV2	FJ496200	C	83.5	90.7	78.3	74.3	81.7	2.01
97ZA012	AF286227	C	83.6	91.2	76.8	73.6	81.3	1.03

### Relating sequence conservation to virus inhibition

By comparing the degree to which immunodominant putative CD8+ epitopes are conserved with corresponding sequences within virus isolates used in the VIA panel and their degree of inhibition, a strong indication that the focusing of immunodominant vaccine-induced responses towards conserved regions of HIV trended towards better global virus inhibition than towards more variable regions (p = <0.0003). The conservation scores of putative immunodominant CD8 epitopes shown in Table S2 (in file S1). By subdividing the viruses that were inhibited into low (1.5–2.5 logs), medium (2.5–3.5 logs) and high (>3.5 logs) levels, data indicates that the more conserved the epitope, the higher the degree of virus inhibition.

**Figure 3:**
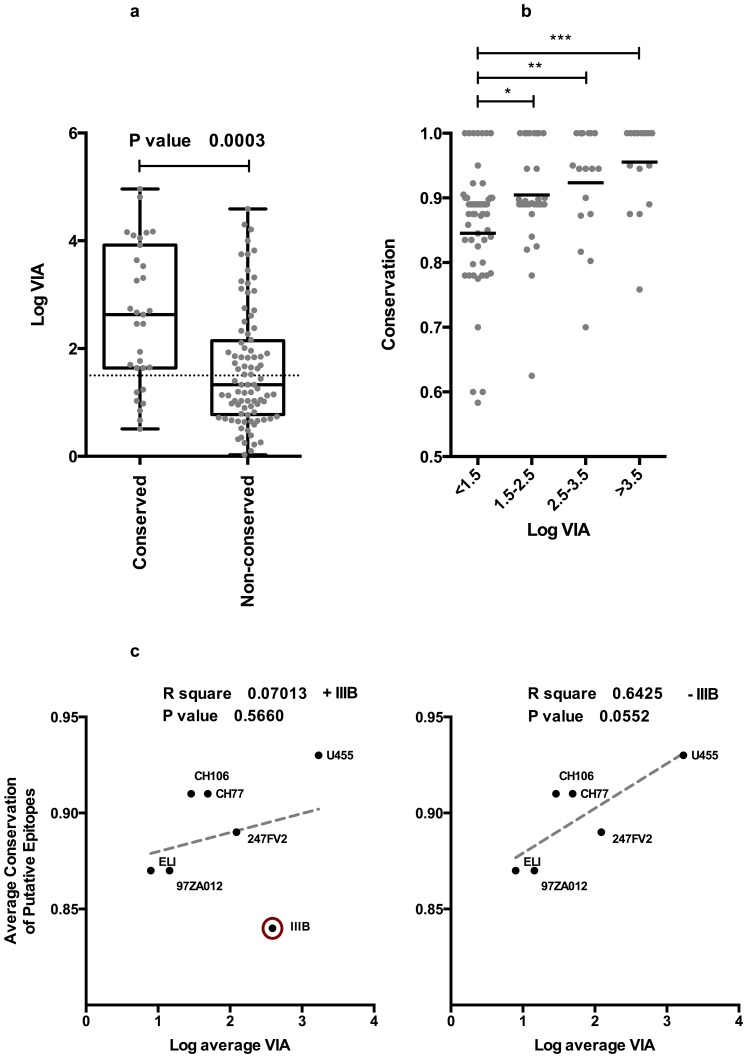
The effect of vaccine-induced CD8 responses targeting conserved regions on *in vitro* virus inhibition. A) box whisker plot comparing the average capacity to inhibit virus replication when targeted sequence is conserved, a Mann-Whitney test for unmatched pairs was used to determine that virus inhibition was significantly higher when vaccines recognized a putative epitope conserved within the relevant virus B) a box whisker plot illustrating that vaccines inhibiting viruses to highest levels recognize epitopes significantly more conserved than those inhibited to lesser levels (Mann Whitney t-test). C) Linear regression analysis relating average conservation of putative epitopes targeted to average inhibition of individual viruses top, IIIB virus is encircled. There is a slight trend towards inhibition of viruses where targeted putative epitopes were more conserved, IIIB is encircled as an outlier (left panel). Where IIIB is excluded (right panel) trend becomes more pronounced and approaches significance.

The fact that the degree of conservation to immunodominant epitopes correlated with virus inhibition in these individuals indicates that there is a quantitative component to this relationship. Furthermore, the median level of virus inhibition where the epitope was entirely conserved was significantly higher than when the epitopes targeted varied (Figure 3a). In addition, the average level of sequence conservation in sequences targeted was related to different levels of virus inhibition (Figure 3b). The average conservation of sequences recognized by individuals unable to inhibit viruses above the 1.5 log cutoff and between 1.5 and 2.5 log was significantly lower than individuals inhibiting viruses above 3.5 log (p = <0.0001), suggesting that high levels of virus inhibition are dependent on high levels of epitope conservation. When the level of conservation in epitopes targeted relative to a specific virus was related to average inhibition of that virus, there was a trend towards higher levels of inhibition of viruses where epitopes targeted are conserved, this trend was only significant when IIIB virus was eliminated from analysis as a result of the possibility of a confounding Nef reading frame (Figure 3c).

## Discussion

Vaccination with Ad35 GRIN/Env-induced broad, polyfunctional responses, predominantly in CD8+ T-cells. Antigen-specific CD8+ T-cell responses were found to be predominantly of a phenotype expressing IFNγ, TNFα and/or CD107a with little or no IL-2 (Figure S5 in file S1) [27]. The expression of Lysosome-associated membrane protein (LAMP), CD107a marks the release of cytolytic granules and has therefore been used as a surrogate for T-cell mediated cytotoxicity [33], a potential mechanism by which the VIA assay is thought to operate. A functional VIA was used to ascertain whether responding subjects were capable of mediating *in vitro* inhibition of a panel of diverse HIV subtypes. To discern whether focusing on particular parts of the virus proteome would better enable inhibition of such viruses, epitope mapping was performed. The total number of responses across the entire vaccine insert in the selected vaccinees ranged from 1–9 epitopes, with a median of 4 epitopes being recognised per subject. The breadth of this response was comparable to that induced by individuals in the STEP trial, where 62% of individuals mounted cellular responses to 2–3 proteins [11], with a median of 1, 1 and 2 subpools for Gag, Nef and Pol, being targeted respectively [8]. The vaccine insert for Groups A–C of this study contained the Env antigen in addition to GRIN, which may have accounted for the increased response breadth in certain individuals. Several putative epitopes within particular antigen pools were found to be preferentially targeted by individuals displaying certain HLA alleles such as the three HLA A*03 volunteers targeting the putative QIYAGIKVK epitope.

The hierarchy of inhibition in the VIA virus panel was U455> IIIB> 247FV2> CH077> CH106> ZA97012> ELI. The improved inhibition of U455 was partly a result of its' high level of overall sequence identity with the clade A/D-derived vaccine insert sequence. However, given that the clade D virus, ELI, was inhibited by CD8+ T cells in fewer vaccinees than the clade B virus IIIB, and yet shared a higher level of % sequence identity with the vaccine insert (Table 3), it is possible that overall sequence conservation alone does not completely account for the relative levels of virus control seen within this group of vaccinees. One potential explanation for the anomalous levels of IIIB inhibition may stem from the defective Nef reading frames in many isolates of this virus [34], which may prevent CTL evasion - through abrogating the down-regulation of HLA A and B alleles loaded with viral epitopes on the surfaces of infected cells, although this was not assessed here.

The majority of vaccinees were found to be capable of inhibiting multiple virus isolates, with two able to inhibit all viruses in the panel to >1.5 logs. A trend towards higher inhibition of viruses, where the targeted epitopes were conserved, was maintained in all vaccinees. Moreover, individuals capable of inhibiting virus replication to high levels (>3.5 logs) targeted responses to significantly more conserved regions than those either unable to inhibit or demonstrated modest levels of virus inhibition (Figure 3b). Unfortunately, the targeting of multiple epitopes of varying levels of conservation relative to viruses used in the VIA panel made it difficult to resolve exactly whether targeting a particular epitope was most likely to result in control of each virus, although in several instances the targeting of one epitope was sufficient to control multiple viruses.

Interestingly, two vaccinees, 048 and 053, mounted immunodominant responses to a sequence within Env containing the putative A*02 and B*3801 epitope, MHEDIISLW. Both were able to inhibit all viruses in this panel and the levels and pattern of their inhibition was similar. Both epitopes were conserved within all virus isolates aside from the CH077 virus, where isoleucine was substituted with valine at position 5. Previous data suggest that targeting the Env antigen by CD8+ T-cell responses in HIV infected individuals was associated with higher viral loads and therefore undesirable [5]. This was mitigated by the possibility that, unlike Gag, *de novo* synthesis of Env during natural infection would be necessary for the intracellular processing of Env required for the generation of Env-specific CD8 T-cell responses, thus delaying the emergence of these populations [5]. However, a pre-existing, response to Env may still be protective in the context of vaccination [35]. This suggests that the sequence targeted is more important than its parent antigen; because sequence diversity in ENV overall is greater than p24 or RT this only reduces the likelihood of targeting conserved regions within this antigen and does not preclude them from being protective.

In several instances virus-specific inhibition could be readily inferred and explained by specific epitopes being targeted and their conservation in the inhibited viruses. Two vaccinees, 036 and 139, possessing the protective B*2703 allele were found to be capable of high level inhibition of viruses containing the KK10 epitope in p24, while viruses containing lysine substitutions previously described were not inhibited ELI (L268V) and 97ZA012 (L268M). Interestingly, neither vaccinee was able to inhibit the CH106 virus despite the presence of the wild type epitope. This may suggest a possible issue in its processing and/or presentation, influencing levels of antigen on the infected cell surface [36], although processing prediction algorithms were unable to predict this.

Together, these data indicate that targeting of immunodominant CD8+ T cell responses towards highly conserved regions of the virus proteome is likely to enable the cross-clade inhibition of viruses and hence be beneficial for any future vaccine strategy, most likely due to the functional importance of any such regions - placing fitness constraints upon their capacity to be varied [37], [38]. Although many current vaccines may be unable to induce responses covering the extensive sequence variation generation generated by HIV, the use of epitope mapping, combined with functional assays, such as VIA, may be a valuable predictor of vaccine efficacy in the future. Sieve analyses of virus sequences isolated from infected STEP trial vaccinees showed that breakthrough viruses to have alterations in the epitopes targeted by T-cell responses of vaccine recipients [39]. Increasing the breadth, coverage and conservation of epitopes recognized following vaccination would potentially reduce the number of breakthrough viruses and consequently, reduce the frequency of infection [39], [40]. Through combining the use of epitope mapping and VIA to further define the immunogenicity of polyvalent vaccine inserts designed to increase response breadth or by eliminating less conserved regions of the HIV proteomes in future vaccine regimens [41], [42], we believe that it may be possible to induce and detect responses capable of inhibiting the majority of circulating virus variants *in vitro* and hence may allow better prediction of *in vivo* efficacy.

Developing vaccines capable of inducing potent anti-viral T cell responses towards highly conserved regions of HIV-1 should be a priority in the rational design of the T-cell-based component/approach to an effective HIV vaccine and such approaches are currently in development [41], [43], [44]. These strategies would preclude the emergence of immunodominant responses to highly variable regions of the virus, which would likely act as immunological decoys and would have little selective impact on the virus. One of these concepts has already entered into Phase I clinical testing in a prime boost combination using novel vector combinations with and without DNA priming [45]. Early pre-clinical data indicate that it is possible to elicit responses to conserved regions through vaccination [46].

Through characterizing which regions targeted by T cell responses have the most structural or functional impact, along with the reconciling the extent of their conservation, rational T cell immunogen design can have maximum impact on the virus and a global application.

## Supporting Information

File S1 **File containing Figures S1–S5, and Tables S1–S3.** Figure S1. Regions recognized within GRIN gag overlaid onto IIIB. Figure S2. Regions recognized within GRIN Pol overlaid onto IIIB. Figure S3. Regions recognized within GRIN Nef overlaid onto IIIB. Figure S4. Regions recognized within GRIN ENV overlaid onto IIIB. Figure S5. Upper panel representative flow plot of vaccine-induced Nef-specific CD8 response. Lower Panel SPICE plots of vaccine induced CD8 responses. Table S1. Peptides mapped in vaccines. Table S2. Conservation Scores of putative CD8 epitopes. Table S3. Inhibition levels and percentages of all viruses used in the VIA panel.
(DOCX)
