# Supplementary material for: Broad HIV Epitope Specificity and Viral Inhibition Induced by Multigenic HIV-1 Adenovirus Subtype 35 Vector Vaccine in Healthy Uninfected Adults
Source: PLoS One. 2014 Mar 7;9(3):e90378. doi: 10.1371/journal.pone.0090378 (PMC3946500; doi:10.1371/journal.pone.0090378)
Supplement: File S1 — File containing Figures S1–S5, and Tables S1–S3. Figure S1. Regions recognized within GRIN gag overlaid onto IIIB. Figure S2. Regions recognized within GRIN Pol overlaid onto IIIB. Figure S3. Regions recognized within GRIN Nef overlaid onto IIIB. Figure S4. Regions recognized within GRIN ENV overlaid onto IIIB. Figure S5. Upper panel representative flow plot of vaccine-induced Nef-specific CD8 response. Lower Panel SPICE plots of vaccine induced CD8 responses. Table S1. Peptides mapped in vaccines. Table S2. Conservation Scores of putative CD8 epitopes. Table S3. Inhibition levels and percentages of all viruses used in the VIA panel. (DOCX) [file pone.0090378.s001.docx]

Figure S1. Regions recognized within GRIN gag overlaid onto IIIB

Figure S2. Regions recognized within GRIN Pol overlaid onto IIIB

Figure S3. Regions recognized within GRIN Nef overlaid onto IIIB

Figure S4. Regions recognized within GRIN ENV overlaid onto IIIB

Figure S5. Upper panel representative flow plot of vaccine-induced Nef-specific CD8 response. Lower Panel SPICE plots of vaccine induced CD8 responses


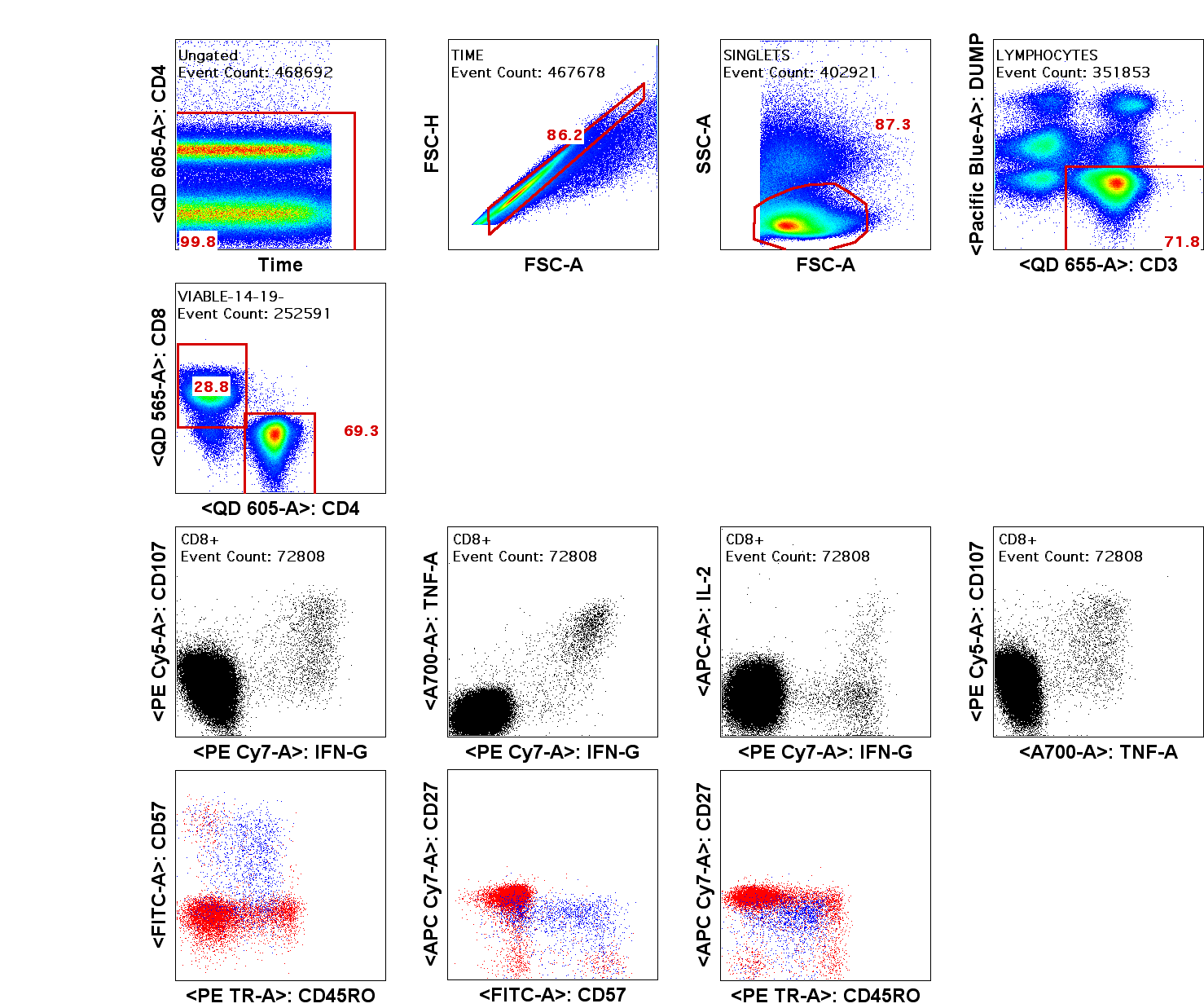


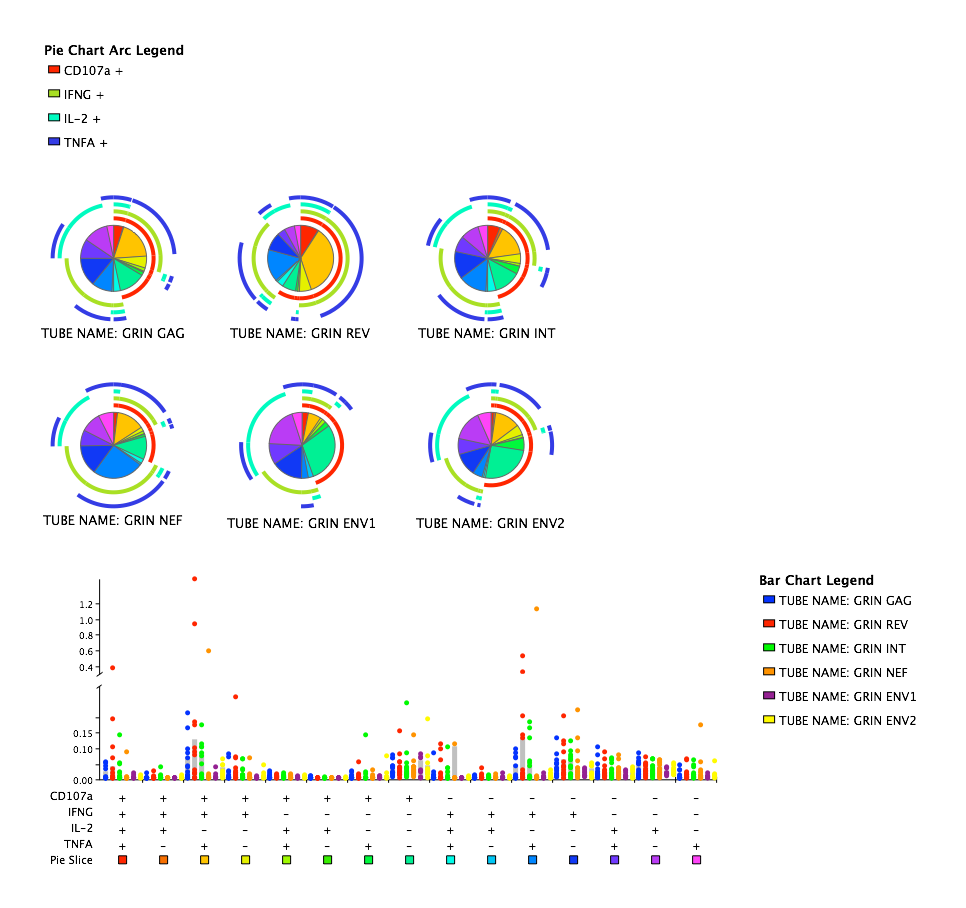


Table S1. Peptides mapped in vaccinees

| PID | Antigen | Peptide |
| --- | --- | --- |
| 005 | GAG | DAWEKIRLRPG |
| 005 | INT | MAVFIHNFKRKGGIG |
| 005 | INT | LQKQITKIQNFRVYYR |
| 005 | NEF | DSRLALKHRAQELHP |
| 005 | ENV | TRKSYRIGPGQTFYA |
| 005 | ENV | PCRIKQIIRMW |
| 010 | ENV | PCRIKQIIRMWQRVG |
| 017 | GAG | DRFALNPSLLETTEG |
| 017 | RT | KWTVQPIMLPDKESW |
| 017 | RT | KVAMESIVIWGKTPK |
| 017 | INT | SGIRKVLFLDG |
| 017 | INT | TSAAVKAACWWANIQ |
| 017 | ENV | PCRIKQIIRMW |
| 018 | INT | FNLPPIVAKEI |
| 025 | RT | STNNETPGVRY |
| 025 | RT | IYAGIKVKQLC |
| 028 | ENV | AVFLGFLGAAG |
| 030 | NEF | REVLIWKFDSRLALK |
| 036 | GAG | YKRWIILGLNK |
| 036 | RT | IYAGIKVKQLC |
| 040 | RT | KGSPAIFQSSM |
| 040 | RT | VQPIMLPDKESW |
| 040 | RT | ESIVIWGKTPK |
| 040 | ENV | EFNMWKNNMVD |
| 040 | ENV | ENIANNAKNIIVQFA |
| 040 | ENV | RAIEAQQQLLK |
| 040 | ENV | AMYAPPIEGVIRCES |
| 040 | ENV | KSYDDIWQNMTWLQW |
| 040 | INT | EFGIPYNPQSQGVVA |
| 048 | RT | EAELELAENRE |
| 048 | RT | QGQDQWTYQIYQ |
| 048 | RT | ESIVIWGKTPK |
| 048 | ENV | MHEDIISLWDQ |
| 048 | ENV | PCRIKQIIRMW |
| 053 | ENV | KQQVYSLFYRLDIEK |
| 053 | ENV | MHEDIISLWDQSLKP |
| 070 | GAG | GATPQDLNVML |
| 070 | ENV | VTVYYGVPVWRDAET |
| 070 | ENV | ENIANNAKNIIVQFA |
| 072 | RT | WASQIYAGIKVKQLC |
| 072 | ENV | KVTFEPIPIHYCAPA |
| 072 | ENV | INCIRPNNNTRKSYRI |
| 072 | ENV | PCRIKQIIRMW |
| 076 | GAG | DRFALNPSLLE |
| 076 | GAG | LFNTVATLYCV |
| 076 | RT | ETFYVDGAANR |
| 076 | INT | PAETGQETAYF |
| 076 | ENV | EFNMWKNNMVDQMHE |
| 076 | ENV | LRKYFSNKTIIFTNS |
| 076 | ENV | SNLLRAIEAQQQLLK |
| 078 | ENV | PGQTFYATDIVGDIR |
| 078 | ENV | PCRIKQIIRMWQRVG |
| 082 | INT | KLVSSGIRKVLFLDG |
| 082 | INT | FNLPPIVAKEI |
| 082 | INT | THLEGKVILVAVHVA |
| 082 | INT | YYRDSRDPIWK |
| 082 | RT | YARKRSAHTNDVRQL |
| 081 | GAG | DRFALNPSLLE |
| 081 | RT | WASQIYAGIKVKQLC |
| 081 | ENV | EFNMWKNNMVD |
| 081 | ENV | RYLRDQQLLGI |
| 085 | GAG | LFNTVATLYCV |
| 085 | GAG | KALRAEQATQDVKGW |
| 085 | GAG | DRFALNPSLLE |
| 085 | RT | IEELRAHLLSW |
| 085 | RT | TYQIYQEPFKNLKTG |
| 085 | RT | WETWWMDYWQATWIPE |
| 085 | INT | KLVSSGIRKVL |
| 085 | INT | EDHERYHSNWR |
| 085 | INT | FRVYYRDSRDPIWK |
| 116 | GAG | DAWEKIRLRPG |
| 116 | GAG | GATPQDLNVML |
| 116 | GAG | VGNIYKRWIILGLNK |
| 116 | NEF | EEEEVGFPVR |
| 116 | NEF | IWKFDSRLALKHRAQELHP |
| 120 | RT | YNVLPQGWKGSPAIF |
| 120 | RT | EVVQKVAMESI |
| 120 | INT | EFGIPYNPQSQGVVA |
| 120 | NEF | EEEEVGFPVR |
| 135 | INT | FNLPPIVAKEI |
| 136 | GAG | EQKDREQVPPLVSLK |
| 136 | RT | ISPIETVPVTL |
| 136 | RT | IEELRAHLLSW |
| 139 | GAG | YKRWIILGLNK |
| 145 | GAG | DAWEKIRLRPG |
| 145 | RT | VHGVYYDPSKDLVAE |
| 145 | NEF | IWKFDSRLALK |
| 145 | RT | WIPEWEFVNTP |
| 154 | GAG | DRFALNPSLLETTEG |
| 154 | RT | GKLNWASQIYAGIKV |
| 154 | INT | TKIQNFRVYYR |
| 154 | NEF | REVLIWKFDSRLALK |

Table S2. Conservation Scores of putative CD8 epitopes

| GRIN | U455 | IIIB | ELI | CH77 | CH106 | 247FV02 | 97ZA012 |
| --- | --- | --- | --- | --- | --- | --- | --- |
| EDHERYHSNW | EDHEKYHCNW | DEHEKYHSNW | EEHEKYHNNW | EEHEKYHNNW | DEHEKYHSNW | EEHEKYHNNW | EDHEKYHSNW |
| Conserved | 0.80 | 0.70 | 0.70 | 0.70 | 0.70 | 0.70 | 1.00 |
| EEVGFPVR | WLEGFPVR | EEVGFPVT | DEVGFPVR | EEVGFPVR | EEVGFPVR | GEVGFPVR | EEVGFPVR |
| Conserved | 0.63 | 0.88 | 0.88 | 1.00 | 1.00 | 0.88 | 1.00 |
| IPYNPQSQGV | IPYNPQSQGV | IPYNPQSQGV | IPYNPQSQGV | IPYNPQSQGV | IPYNPQSQGV | IPYNPQSQGV | IPYNPQSQGV |
| Conserved | 1.00 | 1.00 | 1.00 | 1.00 | 1.00 | 1.00 | 1.00 |
| ETFYVDGAANR | ETFYVDGAANR | ETFYVDGAANR | ETFYVDGAANR | ETFYVDGAASR | ETFYVDGAANR | ETFYVDGAANR | ETFYVDGAANR |
| Conserved | 1.00 | 1.00 | 1.00 | 0.91 | 1.00 | 1.00 | 1.00 |
| LPPIVAKEI | LPPVVAKEI | LPPVVAKEI | LPPVVAKEI | LPPVVAKEI | LPPIVAKEI | LPPIVAKEI | LPPIVAKEI |
| Conserved | 1.00 | 1.00 | 1.00 | 1.00 | 0.89 | 0.89 | 0.89 |
| KIEELRAHL | KIEELRAHL | KIEELRQHL | KIEKLREHL | KIEELRQHL | KIEELREHL | KVEELREHL | KIEDLRQHL |
| Conserved | 1.00 | 0.89 | 0.78 | 0.89 | 0.89 | 0.78 | 0.78 |
| WKFDSRLALK | WKFDSTLALK | ---------- | WRFNSRLAFE | WRFDSRLAFQ | WKFDSRLAFH | WKFDSHLARR | WEFDSSLARR |
| Conserved | 0.90 | 0.00 | 0.60 | 0.70 | 0.80 | 0.70 | 0.60 |
| AEQATQDVKGW | AEQATQDVKNW | AEQASQEVKNW | AEQASQDVKNW | AEQASQEVKNW | AEQASQDVKNW | AEQATQDVKNW | AEQATQEVKNW |
| Conserved | 0.91 | 0.73 | 0.82 | 0.73 | 0.82 | 0.91 | 0.82 |
| SPAIFQSSM | SPSIFQSSM | SPAIFQSSM | SPAIFQSSM | SPAIFQSSM | SPAIFQSSM | SPAIFQSSM | SPAIFQSSM |
| Conserved | 0.89 | 1.00 | 1.00 | 1.00 | 1.00 | 1.00 | 1.00 |
| KVAMESIVIW | KVSTESIVIW | KITTESIVIW | RISTESIVIW | KISTESIVIW | KIAKESIVIW | KIALEGIVIW | KIALESIVIW |
| Conserved | 0.80 | 0.70 | 0.60 | 0.70 | 0.80 | 0.70 | 0.80 |
| LFNTVATLY | LYNTVAVLY | LYNTVATLY | LYNTVATLY | LYNTVAVLY | LFNTVAVLY | LYNTVATLY | LYNTVATLY |
| Conserved | 0.78 | 0.89 | 0.89 | 0.78 | 0.89 | 0.89 | 0.89 |
| MHEDIISLW | MHEDIISLW | MHEDIISLW | MHEDIISLW | MHEDVISLW | MHEDIISLW | MHEDIISLW | MHEDIISLW |
| Conserved | 1.00 | 1.00 | 1.00 | 0.89 | 1.00 | 1.00 | 1.00 |
| GQDQWTYQI | GQDQWTYQI | GQGQWTYQI | GHGQWTYQI | GQDQWTYQI | EQGQWTYQI | GNDQWTYQI | GDDQWTYQI |
| Conserved | 1.00 | 0.89 | 0.78 | 1.00 | 0.78 | 0.89 | 0.89 |
| RYLRDQQL | RYLQDQQL | RYLKDQQL | RYLKDQQL | RYLQDQQL | RYLRDQQL | RYLRDQQL | RYLKDQQL |
| Conserved | 0.88 | 0.88 | 0.88 | 0.88 | 1.00 | 1.00 | 0.88 |
| RAIEAQQQLL | RAIEAQQHLL | RAIEAQQHLL | RAIEAQQHLL | RAIEAQQHLL | RAIEAQQHML | KAIEAQQHML | RAIEAQQHML |
| Conserved | 0.90 | 0.90 | 0.90 | 0.90 | 0.80 | 0.70 | 0.80 |
| NNETPGVRY | NNETPGVRY | NNETPGIRY | NNETPGIRY | NNETPGIRY | NNETPGIRY | NNETPGIRY | NNETPGIRY |
| Conserved | 1.00 | 0.89 | 0.89 | 0.89 | 0.89 | 0.89 | 0.89 |
| NIYKRWII | DIYRRWII | EIYKRWII | EIYKRWII | EIYKRWII | EIYKRWII | DIYKRWII | DIYKRWII |
| Conserved | 0.75 | 0.88 | 0.88 | 0.88 | 0.88 | 0.88 | 0.88 |
| IMLPDKESW | IQLPEKDSW | IVLPEKDSW | IKLPEKESW | IVLPEKDSW | IKLPEKDSW | IKLPEKDSW | IQLPEKDSW |
| Conserved | 0.89 | 0.89 | 0.78 | 0.89 | 0.89 | 0.89 | 0.89 |
| QIYAGIKVK | QIYAGIKVK | QIYPGIKVR | QIYPGIKVR | QIYPGIKIK | QIYAGIKVR | QIYPGIKVK | QIYPGIKVR |
| Conserved | 1.00 | 0.78 | 0.78 | 0.89 | 0.89 | 0.89 | 0.78 |
| KRWIILGLNK | RRWIILGLNK | KRWIILGLNK | KRWIIVGLNK | KRWIILGLNK | KRWIILGLNK | KRWIILGLNK | KRWIIMGLNK |
| Conserved | 0.90 | 1.00 | 0.90 | 1.00 | 1.00 | 1.00 | 0.90 |

Table S3. Inhibition levels and percentages of all viruses used in the VIA panel.

| Virus | Clade | Mean log VIA | Median log VIA | Low log VIA | High log VIA | # inhibited by vaccinees | % inhibited |
| --- | --- | --- | --- | --- | --- | --- | --- |
| U455 | A | 3.10 | 3.28 | 1.12 | 4.3 | 23 | 95.8 |
| IIIB | B | 2.36 | 1.94 | 0.65 | 4.96 | 17 | 70.8 |
| 247FV2 | C | 2.01 | 1.69 | 0.03 | 4.17 | 15 | 62.5 |
| CHO77 | B | 1.77 | 1.64 | 0.39 | 4.81 | 14 | 58.3 |
| CH106 | B | 1.37 | 1.31 | 0.26 | 3.26 | 10 | 41.7 |
| ZA97012 | C | 1.03 | 0.72 | 0 | 4.16 | 5 | 20.8 |
| ELI | D | 0.88 | 0.77 | 0.25 | 2.33 | 3 | 12.5 |
